# Looking at the Data on Smoking and Post-COVID-19 Syndrome—A Literature Review

**DOI:** 10.3390/jpm14010097

**Published:** 2024-01-16

**Authors:** Antigona Carmen Trofor, Daniela Robu Popa, Oana Elena Melinte, Letiția Trofor, Cristina Vicol, Ionela Alina Grosu-Creangă, Radu Adrian Crișan Dabija, Andrei Tudor Cernomaz

**Affiliations:** 1 Discipline of Pneumology, III-rd Medical Department, Faculty of Medicine, University of Medicine and Pharmacy “Grigore T. Popa”, 700115 Iasi, Romania; antigona.trofor@umfiasi.ro (A.C.T.); oana-elena.melinte@umfiasi.ro (O.E.M.); cristina.vicol@umfiasi.ro (C.V.); ionela-alina-i-grosu@d.umfiasi.ro (I.A.G.-C.); radu.dabija@umfiasi.ro (R.A.C.D.); tudor.cernomaz@umfiasi.ro (A.T.C.); 2 Clinical Hospital of Pulmonary Diseases, 700116 Iasi, Romania; 3 Private Practice in Psychiatry, 06800 Nice, France; letitia.trofor@yahoo.com

**Keywords:** post-COVID-19 syndrome, smoking, risk factors

## Abstract

Long COVID is a recently described entity that is responsible for significant morbidity and that has consequences ranging from mild to life-threatening. The underlying mechanisms are not completely understood, and treatment options are currently limited, as existing data focus more on risk factors and predictors. Smoking has been reported as a risk factor for poor outcomes of acute SARS-CoV-2 infection and seems to also play a role in mediating post-COVID-19 symptoms. We aimed to review relevant work addressing the interaction between smoking and long COVID in order to characterize smoking’s role as a risk factor and possibly identify new research directions. Methods: The PubMed/MEDLINE database was searched using the keywords ‘smoking’, ‘long COVID’, and ‘post-acute COVID’ to identify relevant English-language articles published up to October 2023. Results and conclusions: From the 374 initial hits, a total of 36 papers were deemed relevant to the aim of the review. There was significant variability concerning the ways in which tobacco usage was quantified and reported; still, there is compelling evidence linking smoking to an increased risk of developing manifestations of post-acute-COVID disease. Some clinical conditions, such as dyspnea, cardiovascular symptoms, and cognitive or mental-health impairment, seem to be relatively strongly associated with smoking, while the connection between smoking and upper-airway involvement seems less certain. The available data support recommending smoking cessation as a clinical tool for the prevention of long COVID.

## 1. Introduction

Long COVID is a relatively well-known disease state that is generally defined by the presence of symptoms of variable intensity three months following the incomplete resolution of an acute SARS-CoV-2 infection, with those symptoms lasting for at least two months and possibly for more than a year [1]. Time points of four and twelve weeks are used to define a different entity, ongoing COVID-19 [2].

The clinical picture of long COVID is complex and of variable severity, but quality of life is generally impaired. A comprehensive metanalysis that included 57 studies and 250,351 COVID survivors reviewed clinical features [3] of long COVID and classified them into five main groups: neurological (headache, taste/smell disturbances, cognitive impairment, memory and concentration issues), mental-health-related (depression, sleep disorders, generalized anxiety disorder), respiratory (fibrosis, restrictive ventilatory defects, chronic cough, persistent dyspnea), mobility-impairing (decreased exercise tolerance) and general (weight loss, myalgia, pain, fever, fatigue, arthralgia). Various additional symptoms and clinical phenomena have been also considered, and multiple pathophysiological mechanisms have been suggested [2]. These mechanisms include direct viral toxicity, endothelial damage, immune dysregulation, and persistent anomalies of the angiotensin-converting enzyme 2 pathway. All of these mechanisms may be modulated by smoking, either by a direct effect of nicotine or through indirect pathways, such as increased inflammation.

From a pragmatic point of view, clinical researchers have attempted to identify potential risk factors and predictors of disease severity. This task was complicated by the plethora of potential issues covered by the umbrella term “long COVID” and by the limited amount of data usually available in retrospective studies.

Smoking is a well-recognized risk factor for various respiratory and cardiovascular conditions and has complicated effects on the central nervous system. For these reasons, smoking is generally regarded as a risk factor for adverse outcomes in acute forms of SARS-CoV-2 infection, despite some controversies regarding the putative protective role of nicotine. Although smoking status is frequently noted in medical records and is thus available for retrospective reports and analyses, there is no clear consensus regarding its implications for the physiopathology of long COVID.

The aim of this paper is to review available published data in order to assess smoking as a potential predictor of the risk of developing post-acute COVID-19 from a clinical point of view and, potentially, to explore underlying mechanisms.

## 2. Method and Research Design

A MEDLINE/PubMed database literature search was performed using the keywords ‘smoking’, ‘long COVID’, and ‘post acute COVID’ to build the simple logic structure ((long COVID) OR (post-acute COVID) AND (smoking)). No article-type or time restrictions were used (up to October 2023), but the language of publication was limited to English.

**Figure 1:**
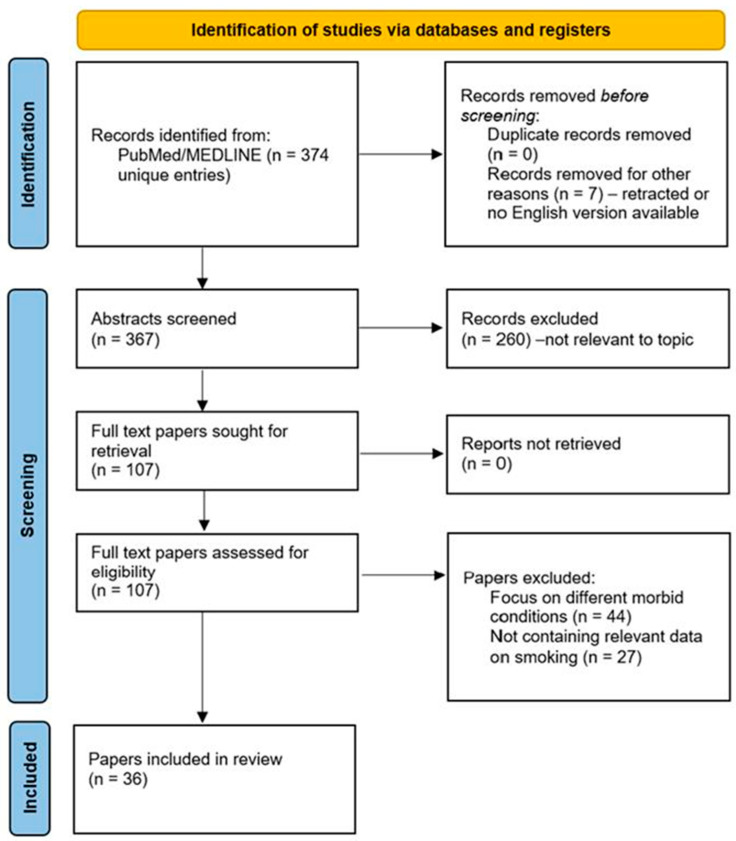
PRISMA flowchart illustrating the article-selection process. Out of 374 potential hits from PubMed/MEDLINE, 36 papers were included in the final analysis.

A total of 374 entries were identified, and the abstract of each entry was screened for relevance. If the abstract screening proved inconclusive, the full article was analyzed for relevance, which was defined as results or conclusions pertaining to smoking as a risk factor/predictor. The article-selection process is illustrated in the PRISMA flowchart (Figure 1).

## 3. Results

After the searching and screening processes were completed, a total of 36 papers containing relevant data on the potential role of smoking as a predictor or risk factor for long COVID symptoms were retained. Relevant titles and details are summarized in Table 1.

A total of twelve articles including over 1 million subjects reported results supporting an association and possibly causative link between smoking and developing long COVID. Furthermore, seven papers reported deleterious effects of smoking on long COVID patients, such as increased number of symptoms, higher severity, late resolution, and greater impact on quality of life.

Some papers reported more specific results concerning the potential harmful effects of smoking on various systems that can be affected by long COVID: three concerning cardiovascular involvement, four about respiratory disorders and persistent pulmonary lesions, six about neurological and mental health impacts and two about other entities.

Among the 34 papers, three were systematic or literature reviews in which smoking was reported to be a potential risk factor or clinical predictor of the risk of developing symptoms of long COVID (possibly specifically in older patients and female patients).

There were five cohort studies that found no clear effect of smoking on the risk of developing long COVID or influence of smoking on the evolution of long COVID symptoms. Two papers reported no specific association between smoking and persistent olfactory and taste disturbances.

Four papers make a clear distinction between current-smoker and former-smoker status when assessing risk; the rest either consider only current smoking status or use an umbrella concept such as smoking history.

There are limited data available on the use of alternative nicotine-containing products; three articles mention vaping, secondhand smoking, or snuff (snus) as having a potential effect. It is worth mentioning that the data on vaping come from a particularly large cohort.

Four articles mention smoking cessation as a potentially useful strategy for managing long COVID in their conclusions.

## 4. Discussion

The relation between COVID-19 and smoking proved complex and difficult to assess, even for acute COVID-19. Although relevant raw data have been published, there are sometimes different interpretations of these data: a 2020 systematic review and meta-analysis of 22 COVID-19 studies found that smoking has a slight detrimental effect on the risks of developing severe disease and of mortality, especially for younger patients who do not have diabetes [40], while a later re-analysis of the same data reached the opposite conclusions, suggesting a potentially protective role for nicotine [41] (albeit taking into account the possibility of a reporting bias and selection bias in the original studies). Current opinion favors a deleterious effect of smoking on outcomes of acute COVID-19 based on experimental [42] and clinical data [43].

Published data paint a hazy picture of the role of smoking in the pathogenesis of long COVID. Some cohorts specifically report no evidence of a link, association or predictive role. It is worth mentioning that a large number of included studies were not designed to highlight such an effect, as they aimed to match the cohort under study and the controls in terms of smoking status.

Still there are published studies that identify correlations between smoking and various aspects of long COVID.

Smoking in the month preceding SARS-CoV-2 infection was reported as the most important predictor of the risk of developing long COVID symptoms (mainly those relevant to capacity for self-care). Smoking was followed in importance by poor sleep quality and low physical activity in a British longitudinal study [28] that included 1581 patients.

Similarly, smoking and vaping are reported alongside female gender, obesity, older age, low-income household, and being a healthcare worker as predictors for persistent COVID-19 symptoms at the 12-week milestone in a large British community study that included over 600,000 subjects [14].

Post-COVID-19 manifestations may have serious consequences, apart from lower quality of life: one analysis using the OnCovid registry data on 2634 cancer patients [8] reported an increased risk of death (hazard ratio 1.80, 95% CI 1.18–2.75) and listed smoking history as a potential risk factor.

The functional impact of persistent COVID-19 symptoms may be difficult to assess objectively, but instruments have been developed for this purpose. One such instrument is the Post-COVID-19 Functional Status scale [44]; using this tool with an Egyptian cohort found a prognostic role for smoking [12].

Data from a Japanese long COVID cohort study that tested various therapies suggest that respiratory manifestations are the most prevalent and usually the most aendable to medical interventions. The study data also support the idea that smoking may increase the recovery period for specific symptoms [20].

Some published data suggest a predictive role for smoking across all groups of symptoms. For example, a Bangladeshi cohort study [21] reports odds ratios for smokers vs non-smokers of 1.76 (0.93, 3.34) for developing long COVID, 1.49 (0.92, 2.44) for respiratory symptoms, 1.29 (0.79, 2.11) for cardiovascular problems, 1.69 (1.05, 2.73) for neurological symptoms, and 1.35 (0.70, 2.60) for mental health issues. The generality of these effects may support the hypothesis that persistent inflammation is a cause of long COVID. Along this line of reasoning, another study including 121 mild COVID-19 cases with persistent clinical features found an association between long COVID and low-grade inflammation (instrumentalized as neutrophil count, fibrinogen level, C-reactive protein level and neutrophil/lymphocyte ratio) and reported significant differences between genders [45].

There are also published data focusing on specific symptoms. For example, a Saudi Arabian cohort study investigating persistent taste and smell issues among 2497 COVID patients [33] identified different predictors on stratifying for gender: females had a higher risk overall, particularly for the first SARS-CoV-2 episode, while male gender was associated with smoking and admission to intensive care.

Similarly, in a Chinese cohort of 121 post-COVID-19 patients, persistent chronic cough [34] was linked to current smoking OR 6.95, 95% CI: 1.46–33.14. As a caveat, according to the authors’ interpretation, the unusually large odds ratio may be partially explained by the relatively small number of smokers included in the analysis.

Prolonged respiratory symptoms are a common clinical manifestation of long COVID. Multiple underpinning pathophysiological changes may be responsible. For example, persistent lung lesions, secondary fibrosis, respiratory muscle impairment, and decreased transfer capacity were all reported during the convalescence phase of COVID-19 for more than 50% of patients [46].

Nicotine was reported as having complex effects on the renin–angiotensin system. In terms of its relevance to COVID-19, there are data suggesting down-regulation of the angiotensin 2 (ACE2) receptor in both its membrane-bound and soluble forms [47], but there are conflicting reports on its increased expression in the alveolar and bronchial epithelia of smokers and COPD patients [48]. This change is particularly significant for subjects with COPD stage III and IV, compared with patients with less serious forms of COPD and smokers without airflow limitations. This variable effect of nicotine exposure on ACE2 receptor expression might explain the various contradictory reports on a putative protective role of smoking against COVID-19; furthermore, there are experimental data suggesting that chronic and acute exposure to cigarette smoking have different effects on ACE2 expression [49].

A retrospective study that compared computed tomodensitometry data between 77 long COVID patients and matched controls reported an approximately 10% decrease in lung volume among COVID-19 patients in the absence of clear lung lesions. The authors advance the hypothesis of microfibrotic lesions, possibly linked to prolonged inflammatory changes [50]. This inflammatory hypothesis is supported by other work [51] reporting transfer impairment as the most frequent anomaly and identifying three risk factors: age, disease severity and intensity of systemic inflammation. There are convincing data supporting smoking as an independent risk factor for the persistence of chest X-ray anomalies at 12 weeks after an acute SARS-CoV-2 infection for at least some subgroups of hospitalized patients [37].

Lung fibrosis following pulmonary viral infection is a well-known phenomenon. A variable degree of fibrosis is common; a literature review [52] that included 2018 COVID-19 survivors from 6 studies reported a 44.9% prevalence of fibrotic lesions and found an association with coughing, dyspnea, chest pain, myalgia, and fatigue. The authors identified a series of prognostic factors: computed tomography scores over 18, admission to intensive care, and mechanical ventilation. Somewhat counter-intuitively, COPD is reported as a risk factor, while smoking was not found to have a significant impact. Similarly, a study aimed at identifying risk factors for developing post-COVID-19 lung fibrosis, which followed 387 patients [53], found no significant role for smoking but found significant roles for male gender, need for ventilatory support, persistent breathlessness, and high levels of cytokine-storm markers. The processes underlying of SARS-CoV-2-induced lung fibrosis are not completely understood, but imbalances of the renin–angiotensin system [54] and low levels of circulating interferon gamma [55] have been reported as potentially relevant to fibrosis following COVID-19. Smoking is known to have similar effects [56] but also has a variable effect [57] on the transforming growth factor pathway [58], which is central to fibroblast proliferation and collagen production. However, smaller studies have found a potential negative effect of smoking on post-COVID-19 lung fibrosis [59]. This effect may reflect an indirect influence, as smoking and asthma were good predictors of severe disease and the need for mechanical ventilation.

Muscle mass and capacity are increasingly used as survival predictors of chronic cardiovascular and respiratory obstructive disorders; respiratory-muscle dysfunction may partially explain the restrictive respiratory patterns reported in COVID-19 patients [60]. A metanalysis and literature review attempting to characterize sarcopenia as a risk factor among older adults [61] found a plethora of demographic, socio-economic, and morbid conditions, many of which also been identified as risk factors for long COVID. A cross-sectional study on the body composition of 711 COVID patients reported associations between dynapenia and sarcopenia and lower lung function [62]. The association between sarcopenia and smoking is well known [63], as extensive data have been collected from COPD patients [64]; the underlying mechanisms are complex and incompletely elucidated, but a role has been postulated for systemic inflammation—TNF alpha, IL1, IL6, and C-reactive protein have been investigated in this context [65].

Considering the available evidence, one may assume that at least some of the respiratory manifestations of long COVID are consequences of multiple pathophysiological alterations, including but probably not limited to restrictive ventilatory defects in the context of fibrotic lesions of variable severity that developed as a consequence of prolonged inflammation and that are potentially aggravated by respiratory muscle disfunction. Smoking has a well-known role in prolonging and aggravating inflammatory events, and this role may be partially explained by the nicotine–ACE2 interaction. Setting nicotine aside, there are limited data concerning the role of the over 2500 known components of tobacco smoke in COVID pathogenesis.

Along the same line, dyspnea and fatigability may reflect multisystemic involvement (typically respiratory and cardiovascular involvement), as the empiric data suggests. For example, one study including 89 COVID-19 patients reported associations between long-term pulmonary dysfunction and age, hypertension, and insulin resistance. This study also identifies relevant biological changes, such as high platelet counts and CXCL9 levels [66]. CXCL9 is a cytokine involved in chemotaxis and lymphocytic migration and has also been investigated as a potential biomarker of heart failure [67]. CXCL9 has been reported to be upregulated in long-term smokers and ex-smokers [68]. This association may underline a link between smoking and respiratory manifestations of long COVID, such as dyspnea and fatigue.

While cardiovascular impairment may explain some symptoms, such as dyspnea or fatigue, new-onset cardiovascular disease was clearly linked to SARS-CoV-2 infection [69], and persistence after 12 weeks is not uncommon. Furthermore, there are published data suggesting an association between smoking and long COVID-related hypertension and tachycardia [16]. Results from a Polish cohort included increased arterial stiffness in COVID-19 convalescents [70], an effect that persisted after controlling for age, sex, body mass index, diabetes and smoking; it is to be expected that smoking in this case will have an additive effect.

Although smoking is known to have a deleterious effect on smell and taste capabilities [71], there are reports suggesting no effect on similar deficiencies related to long COVID. Two small cohorts [23] of previously hospitalized patients found no significant impact of smoking on the risk of developing such symptoms [38], and a larger study found that smoking, male gender and a history of intensive care were correlated with an increased risk of developing dysgeusia [33]. Such contradictory results are difficult to reconcile, but variability in ACE2 expression on the upper-respiratory-tract mucosa may explain the differences: it is lower in younger [72] and female subjects, which are the main demographics associated with better outcomes for acute SARS-CoV-2 infection and which are less likely to be found in a hospitalized cohort.

Regarding cognitive impairment, data from a retrospective Swiss cohort of outpatients (with mild and moderate forms of COVID-19) suggest that more than half of SARS-CoV-2-positive patients reported persistent cognitive symptoms up to 10 months after the acute infection. Specifically, smoking seems to be significantly related to memory impairment [9].

A study that included 54 post-COVID-19 patients with mild neurocognitive impairment [73] found significantly decreased serum levels of brain-derived neurotrophic factor (BDNF) compared with healthy controls. Smoking, or at least nicotine exposure, has a known effect on the BDNF pathway [74], influencing serum levels and possibly the expression of TrkB receptors. Although smoking is generally associated that smoking with higher BDNF levels, recent data show a more nuanced influence—current heavy smoking [75] and total number of smoking [76] years seem particularly important, while light smoking has no significant inductive effect.

Various, non-mutual, exclusive mechanisms have been investigated to explain long COVID manifestations. These mechanisms include viral persistence, immune abnormality, vascular anomalies and autonomic dysregulation. At least for neurological complaints, the available published data seem contradictory; some authors have suggested the possibility of viral persistence in neuronal structures [77], while a study on 25 patients with post-COVID-19 cognitive symptoms [78] found no evidence of persistent viral components or inflammatory markers in cerebrospinal fluid or serum.

Some neurological manifestations of long COVID might be explained by reduced level of serotonin; a recent work [79] explored this hypothesis and postulated a role for interferon-driven inflammation, which could act by decreasing tryptophan absorption and increasing serotonin turnover.

Mental-health effects may be difficult to assess in long COVID patients, and the underlying mechanisms are probably complex; there are data suggesting an association between smoking and sedative use [15] (significant for female gender), a relationship that might not be casual but that rather might reflect anxious behavior. Furthermore, the SARS-CoV-2 epidemic seems to have had an indirect effect on mental health and even somatic symptoms, as was shown in a Greek cohort [80]; similarly, a Turkish transverse study reported that smoking predicted the levels of anxiety experienced by recovered COVID patients who had been hospitalized (OR 4, 95% CI 1.2–12.5) [36], but the authors hypothesize that tobacco use is a coping strategy, rather than a cause. Smoking was found to be associated with sleep disorders, anxiety, and depression in a Chinese cohort that enrolled previously hospitalized COVID-19 patients [39] one year after their discharge, and various mental-health conditions are known to be associated with chronic respiratory diseases [81].

Immune dysregulation has also been discussed as a potential underlying mechanism for persistent COVID-19 symptoms—a hypothesis supported by compelling data showing a higher incidence of autoimmune disorders [82] following the SARS-CoV-2 epidemic. There is no easy way to explore the potential influence of smoking on complex immune responses, as many effects of nicotine are dose-dependent [83], but empiric data show weaker antibody responses in smoking patients [84] and possibly an increased probability of subsequent reinfections [85].

The analysis of published results encountered some methodological limitations: the majority of published studies use a dichotomic (sometimes three-layered) stratification, with smoker/ex-smoker and non-smoker categories; additionally, loosely defined terms such as “light smoking” or “current smoker” are sometimes utilized; finally, there generally seems to be no data available on the intensity of tobacco consumption (expressed as pack-years or otherwise).

Additionally, social and economic status, cultural characteristics, and mental state are a few factors that may function as confounders of the role of smoking in long COVID.

The majority of the studies analyzed have retrospective designs, which limits their power and scope. This limitation was to be expected, given the chronic nature of long COVID. Some of the data were obtained by telephone surveys and various self-reporting tools, an approach that may introduce reporting bias. The broad definition of long COVID may also lead to overdiagnosis, as some symptoms are not specific and the criterion that symptoms ‘should not be explained by other chronic or intercurrent disorders’ (according to the generally accepted definition of long COVID) might not translate easily to clinical or research practice, especially when self-reporting or phone surveys are employed.

Along this line of reasoning, we may infer that the relationship between smoking and the SARS-CoV-2 epidemic does not stop at the clinical level. There are some data suggesting COVID-19 and related social and healthcare policies had a mixed effect on smoking, with some individuals starting or restarting smoking as a coping mechanism and policies encouraging quitting or switching to vaping as a potentially less harmful alternative [86]. Data from a small Turkish cohort suggest the disease itself may play a role as a motivator encouraging smoking cessation and perhaps preventing relapse, especially for patients who believe that smoking has a negative health impact [87]. From the public-health point of view, policies implemented to manage COVID-19 in South Asia [88] were reported to have a positive effect on tobacco and alcohol consumption that differed between genders, a result that may underline the importance of local customs, education levels and socio-economic status. On the other hand, a similar report from Greece [89] found no significant impact of the SARS-CoV-2 epidemic on tobacco usage; such data may support the importance of considering cultural and socio-economic background when assessing smoking on a population level.

The relationship between smoking and COVID-19 seems to be complicated and multifaceted. For example, a British survey of 3179 adults [90] showed that smokers and longtime ex-smokers were more likely to self-report COVID-19, compared to never smokers. The authors suggest differences in hygiene habits, such as washing hands after smoking or vaping, as potential explanations; still, such results underline potential sources of reporting bias when self-reporting methods are employed. Along the same line, the authors of a Turkish study on chronic COVID-19 symptoms [35] suggest that perceived low social economic status is associated with an increase in reported symptomatology, although the underlying mechanism is not elaborated upon.

There are few studies that include recommendations of smoking cessation for long COVID patients. The data may be scant and limited, but given the agreement with general public-health policies, such a recommendation seems like a sensible approach.

There is significant variability in the incidence of various symptoms associated with long COVID when comparing either percentages or rankings; this variability is probably explained by differences between cohorts, methods of gathering data, timing of assessments, and, possibly, differences in the definitions utilized.

There are practically no data that may elucidate the roles of different components of tobacco smoke; clinical data makes use of the umbrella term “smoking”, and fundamental research usually focuses on biological effects of nicotine. With this limitation in mind, we also consider a small number of studies that found similar effects for smoking and vaping [14] and snuff use [24]; this agreement may suggest a central role for nicotine exposure as a risk factor for developing long COVID, considering that the chemical composition of these products is otherwise quite different. Future research initiatives might benefit from including vaping and heated tobacco products in standard data collection, considering the societal changes in tobacco-related habits.

There are some initiatives attempting to address some of these issues and to assess long COVID from a prospective point of view. One such initiative is a recently published protocol [91] attempting to characterize vascular and endothelial anomalies that also includes a systematic tool for the collection of smoking data.

This review has some obvious limitations, at least some of which are external and linked to the nature of the condition under study.

More than one label or keyword may be used for the clinical entity under study. Although both ‘long COVID’ and ‘post acute COVID’ were used as keywords in an attempt to be comprehensive, there might nonetheless be papers using a different or alternative terminology. This possibility may be relevant especially for early papers published before the terms were coined.

Post-acute COVID has a clear but general definition, which may lead to overdiagnosis. Additionally, the intensity and impact of the symptoms are not always reported or available, which limits the value of intergroup comparisons.

The majority of data seem to be either retrospective or generated by bias-prone methods such as phone surveys or self-reporting of symptoms.

Reported smoking data are frequently limited (ex., no data on total tobacco consumption or quit time), which makes it difficult to investigate a dose–response relationship.

## 5. Conclusions

There is compelling evidence to link smoking to an increased risk of developing post-acute COVID manifestations.

There seems to be a relatively strong association between smoking and some definitive clinical components of long COVID, mainly respiratory and heart-related issues; the association with olfactory or taste disturbances seems weak, although selection bias could not be excluded. Smoking probably plays an etiologic role in the underlying physiological phenomena of respiratory, cardiovascular, and, possibly, neurological manifestations of long COVID; the relationship between smoking and at least some cognitive or mental-health-related symptoms may in part be explained by association, rather than causation. Despite limited data, recommending smoking cessation as a clinical tool for prevention of long COVID seems to be a reasonable approach.

Available data on long COVID may be biased, given the retrospective nature of the studies and the sometimes vague definitions utilized; similarly, only limited data on smoking are available. Future research could benefit from using a robust approach to data gathering, such as employing standardized definitions and data types.

## Figures and Tables

**Table 1:** Articles identified using the search terms ‘smoking’, ‘long COVID’ and ‘post acute COVID’ in the MEDLINE/PubMed database and confirmed as containing data relevant to the potential impact of smoking on persistent symptoms associated with SARS-CoV-2; the order is based on search-engine output, using relevance as a criterion.

First Author	Year Published	Type (Cohort Study Unless Otherwise Specified)	Cohort Size	Results/Conclusions
Subramanian A et al. [4]	2022		486,149	smoking and former smoking, high BMI, and a wide range of comorbidities were all associated with an increased risk of reporting symptoms ≥12 weeks after infection
Lippi G et al. [5]	2023	review		cigarette smoking (OR, 1.26; 95% CI, 1.04–1.54) as a clinical predictor of the risk of developing long COVID
Bai F et al. [6]	2022		377	active smoking (AOR 0.19 for former smokers vs. active smokers, 95% CI 0.06–0.62, *p* = 0.002) were also associated with a higher risk of long COVID
Wong MC et al. [7]	2023		2712	smoking associated with severe long COVID (OR = 1.55, 95% CI 1.17–2.05)
Pinato DJ et al. [8]	2021		2634 cancer patients	patients with a history of smoking (vs. no smoking history; *p* = 0.0004) at higher risk of developing sequelae
Desgranges F et al. [9]	2022		418	predictor of memory impairment associated with long COVID
Conti V et al. [10]	2023	review		active smoking, older age and female gender associated with higher risk of developing post-COVID-19 syndrome
Muzyka I et al. [11]	2023		332	unclear effect
Mohamed Hussein AA et al. [12]	2021		444	smoking status has a detrimental effect on pulmonary function (PCFS scale); assessment at 35.31 ± 18.75 days after symptom onset
Wang C et al. [13]	2023	review		smoking possibly associated with an increased risk of developing symptoms of post-acute COVID-19 syndrome
Whitaker M et al. [14]	2022		508,707 + 97,727	smoking and vaping associated with persistence of one or more symptoms for 12 weeks or more (some models of multivariable analysis)
Carrasco-Garrido P et al. [15]	2022		391	higher benzodiazepine and Z-hypnotics use among females with long COVID after stratifying for alcohol and tobacco use
Barthélémy H et al. [16]	2022		956	smoking found to be a predictor of the risk of cutaneous manifestations (OR = 2.34; 95% CI: 1.39–3.92) and tachycardia/hypertension (OR = 2.05; 95% CI: 1.2–3.47); assessment after more than 60 days from onset of symptoms; smoking cessation recommended
Jacobs ET, et al. [17]	2023		1224	no significant effect of smoking history on risk of developing post-acute COVID-19
Román-Montes CM et al. [18]	2023		246	smoking more prevalent among post-COVID-19-syndrome patients; no significant association with dyspnea
Wu Q et al. [19]	2022		308	no predictive role for current smoking status
Takakura K et al. [20]	2023		286	improvement of long COVID may be delayed by smoking; smoking cessation recommended
Afroze F et al. [21]	2022		362	various comorbidities and smoking status are considered independent risk factors for developing neurological and cardiovascular manifestations
Buonsenso D et al. [22]	2022		155	risk factor for not resuming work among long COVID patients (OR 4.106, CI (0.4–11.9), smoking cessation recommended, anxiety more prevalent among female patients
Tarifi A et al. [23]	2021		86	no significant effect on smell or taste, possibly due to small cohort size
Kisiel MA et al. [24]	2023		401 + 98 + 85	smoking and snuff use associated with higher post-COVID-19 symptomatology scores
Chathoth AT et al. [25]	2023		938	smoking considered a significant predictor of the risk of limited functional status associated with post-COVID-19 syndrome
Mclaughlin M et al. [26]	2023		253	greater number of symptoms reported by smokers vs non-smokers or ex-smokers
Tene L et al. [27]	2023		180,759	long COVID associated with smoking (OR = 1.532; 95% CI: 1.358–1.727)
Paul E et al. [28]	2022		1581	smokers and ex-smokers with long COVID at higher risk for experiencing self-care-related difficulties; smoking cessation recommended
Chilunga FP et al. [29]	2023		1886	no clear role; possible ethnic differences
Bamps L et al. [30]	2023		1598	smoking associated with higher risk of long COVID
Vásconez-González J et al. [31]	2023		457	smoking associated with higher risk of developing persistent fatigue in pregnant women with long COVID
Akinci Ozyurek B et al. [32]	2021		315	no significant role of smoking; assessment one month after onset of symptoms
Hennawi YB et al. [33]	2023		2497	smoking associated with significantly longer duration of ageusia
Chen Y et al. [34]	2022		121	smoking associated with higher risk of chronic cough (OR 6.95 95% CI: 1.46–33.14); secondhand smoking is mentioned
Emecen AN et al. [35]	2023		5610	current smoking associated with increased self-reporting of chronic symptoms (OR 1.15, 95% CI: 1.02–1.29)
Cansel N et al. [36]	2021		102	smoking is associated with higher risk of moderate or severe anxiety (OR, 4, 95% CI 1.2–12.5) and higher risk of moderate or severe depression (OR, 8.8, 95% CI 2.5–30.8)
Wallis TJM et al. [37]	2021		101	smoking status reported as an independent predictor of the risk of chest X-ray anomaly at 12 weeks after recovery from acute SARS-CoV-2
Tan HQM et al. [38]	2022		150	no role for smoking in the risk of developing persistent olfactory/taste impairment
Li Z et al. [39]	2023		535	smoking associated with poor sleep quality (OR 2.005, 95% CI 1.044–3.850), anxiety (OR 4.491, 95% CI 2.276–8.861), and depression (OR 5.459, 95% CI 2.651–11.239)

## Data Availability

No new data were created or analyzed in this study. Data sharing is not applicable to this article.
